# Tracking ink composition on Herculaneum papyrus scrolls: quantification and speciation of lead by X-ray based techniques and Monte Carlo simulations

**DOI:** 10.1038/srep20763

**Published:** 2016-02-08

**Authors:** Pieter Tack, Marine Cotte, Stephen Bauters, Emmanuel Brun, Dipanjan Banerjee, Wim Bras, Claudio Ferrero, Daniel Delattre, Vito Mocella, Laszlo Vincze

**Affiliations:** 1 Analytical Chemistry, Ghent University, Krijgslaan 281 S12, 9000 Ghent, Belgium; 2 The European Synchrotron, 71 av des martyrs 38000 Grenoble, France; 3 Laboratoire d’Archéologie Moléculaire et Structurale, CNRS-UPMC, UMR 8220, 4 Place Jussieu, 75005 Paris, France; 4 Inserm & Université Joseph Fourier, Grenoble Institut des Neurosciences, U836 & UMR-S836, 38043 Grenoble, France; 5 CNRS-IRHT-Institut de Recherche et d’Histoire des Textes, 10 rue Molitor, 75016 Paris, France; 6 CNR-IMM-Istituto per la Microelettronica e Microsistemi-Unità di Napoli, via P. Castellino 111, 80131 Napoli, Italy

## Abstract

The writing in carbonized Herculaneum scrolls, covered and preserved by the pyroclastic events of the Vesuvius in 79 AD, was recently revealed using X-ray phase-contrast tomography, without the need of unrolling the sensitive scrolls. Unfortunately, some of the text is difficult to read due to the interference of the papyrus fibers crossing the written text vertically and horizontally. Recently, lead was found as an elemental constituent in the writing, rendering the text more clearly readable when monitoring the lead X-ray fluorescence signal. Here, several hypotheses are postulated for the origin and state of lead in the papyrus writing. Multi-scale X-ray fluorescence micro-imaging, Monte Carlo quantification and X-ray absorption microspectroscopy experiments are used to provide additional information on the ink composition, in an attempt to determine the origin of the lead in the Herculaneum scrolls and validate the postulated hypotheses.

Recently, the writing in rolled Herculaneum papyri was successfully explored by X-ray phase-contrast tomography^1^,^2^. These scrolls are part of a complete library, containing hundreds of such papyrus scrolls. This library was discovered between 1752 and 1754 in the small city of Herculaneum, which was covered and preserved by layers of volcanic material in 79 AD^3^,^4^,^5^,^6^. This is the only library passed on from Antiquity, and is therefore an inestimable treasure. Due to the pyroclastic events associated with the eruption, the scrolls were carbonized and locked-up, rendering the writing on the scrolls unreadable. Previously, it was attempted to read these scrolls by mechanically unrolling them^7^. These attempts, however, usually led to the irretrievable loss of large parts of the text due to the brittle nature of the carbonized scrolls.

As demonstrated by the work of Mocella *et al.*, the writing in the scrolls can now be, at least partially, recovered by non-destructive X-ray imaging methods, without the need of unrolling the scrolls^1^. The readability of the text is however not optimal due to interference of the papyrus fibres, running vertically and horizontally across the document. Recently, Brun *et al.* showed the presence of lead in the papyrus writing, rendering the text more readable when monitoring the Pb X-ray fluorescence (XRF) signal [Brun *et al.*, submitted].

Several hypotheses to explain the presence of Pb and other elements in the sample are assessed using a sequence of non-destructive, X-ray based analytical techniques, providing further insights in the production and history of the Herculaneum manuscripts.

Lead could have been introduced unknowingly, or on purpose.

- An initial hypothesis is Pb contamination of the water used as a solvent for the ink.

- A second hypothesis states that Pb could be present as a contaminant from a bronze container in which the ink was stored, as discussed by Canevali *et al.*^8^.

- Alternatively, Pb could have been knowingly introduced to the ink in a controlled fashion. Lead-based pigments, being a black mineral galena (PbS) or lead white (different mixtures of cerusite (PbCO_3_) and hydrocerusite (2PbCO_3_,Pb(OH)_2_)), were frequently used in ancient times as a pigment for cosmetic products^9^,^10^,^11^. Galena has been proposed as a pigment in black inks in Egyptian papyrus before^12^, whereas minium (Pb_2_^2+^Pb^4+^O_4_) has been reported as a red pigment in Roman writing^13^,^14^.

- Additionally, Pb could originate from a binding medium in the ink: Pb compounds have been used extensively as dryers in paintings as they speed up the process of oil drying^15^,^16^. The use of litharge (PbO) as oil drier is already mentioned by Galen at the 2^nd^ C. A.D. and by Marcellus at the 4^th^ C. A.D^17^,^18^. Furthermore, the use of a lipid-based ink to draft the papyrus writing can be hypothesized^8^,^19^,^20^.

## Results and Discussion

**Figure 1:**
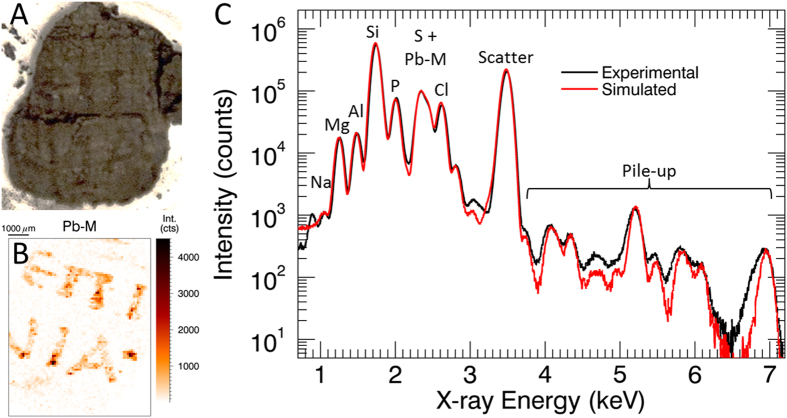
Contrast and brightness enhanced photograph (**A**) and Pb-M XRF signal (**B**) of the large papyrus fragment. The XRF spectra of the writing (**C**) on the large papyrus fragment (Experimental, black curve) and Monte Carlo simulation (Simulated, red curve) using the XMI-MSIM software.

To be able to (dis)prove these hypotheses, two fragments (referred to as the “large” and “small” fragments) from unrolled scrolls were investigated. The two fragments are flat pieces of papyrus, made of different sheets, and presenting E, Π, Ι, N, Ι, Α and O, Λ letters on their surface, respectively. A picture of the large sample is shown in Fig. 1A. In terms of analytical techniques, considering the precious nature of the samples, priority was given to non-destructive imaging methods. Besides, the heterogeneity of the samples at both micro- and millimetric scales motivated the implementation of a multi-scale analysis. Accordingly, macro and micro XRF and X-ray absorption spectroscopy (XAS) techniques were used, at ID21^21^ and BM26A^22^ of the European Synchrotron Radiation Facility (Grenoble, France). In order to have a good statistic record of XRF data in and out of the ink, XRF 2D maps were acquired over the full surface of the samples, with a sub-millimetre beam. As detailed in Brun *et al.* [submitted], the XRF map of Pb perfectly matches with the ink distribution (Fig. 1B) as the writing, faintly visible in Fig. 1A as well, is revealed. From this map, two average XRF spectra were extracted, by summing the pixel spectra corresponding to i) the writing using K-means clustering^23^ and visually comparing the clusters to the XRF distribution images, ii) the papyrus alone, excluding the written areas. The qualitative comparison of these two spectra reveals an increased concentration in Pb, Al and P in the written area, which is also observable on the XRF map (Supplementary Figure S1).

In order to quantitatively determine the ink composition, XRF results were simulated by means of Monte Carlo calculations^24^,^25^,^26^. These simulations model all relevant photon-matter interactions, simulating the trajectories of a large number of photons originating from an X-ray source and undergoing interactions in the sample, to the point of possible detection in a detector. The occurrence of each photon-matter interaction (photoelectric effect/fluorescence, Rayleigh and Compton scattering) as well as direction of propagation and distance between interactions of the photons are simulated. By iteratively comparing the simulated spectrum to its experimental equivalent and adjusting the simulated sample composition after each iteration, the experimental sample composition is determined.

**Figure 2:**
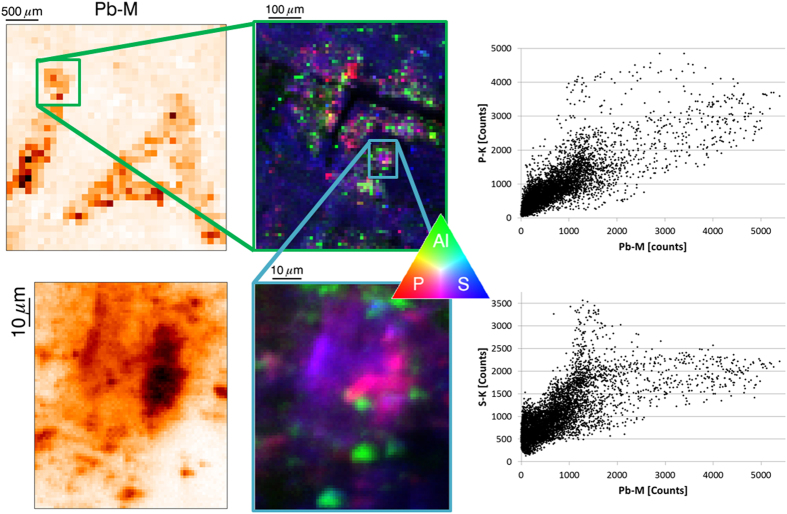
Micro-XRF Pb elemental distribution images on macroscopic (top left, 100 μm step size) to microscopic (bottom left, 1 μm step size) level. Red (P) Green (Al) Blue (S) images are displayed in the centre for different zoom modes (top right: 10 μm step size, bottom right: 1 μm step size) to display the co-localization of Al, P and S with Pb. Pb-P and Pb-S correlation plots are displayed to the right to show the correlation of Pb with P and partial correlation with S.

Figure 1C shows the result of the Monte Carlo simulation based XRF spectrum. A clear correspondence is seen between the experimental spectrum and the theoretical simulation. The main differences are attributed to the lack of simulation for X-rays below 1 keV^25^ and a slight underestimation of the generated pulse pile-up (3.05 keV and >3.5 keV) due to the uncertainty on the simulated detector pulse width. Elemental concentrations are shown in Table 1. Both fragments show a similar C and Si content. The writing on the large fragment contains significantly more Pb, and slightly more Na, Al, P and Cl. The S concentration is lower in the large fragment than in the small one. When comparing the quantification results of the writing to the papyrus of the same fragment, a characteristic increase in Al, P, Pb and in a lesser amount Cl is perceived, showing these elements are clearly associated with the writing. This was further investigated using μXRF, as discussed below (Fig. 2). Additionally, the papyrus in both fragments has a fairly similar composition, only Na and S quantities seem to vary. It should be noted that these quantification results do not represent the composition of the pure ink, but rather represent a mixture of the ink and the underlying papyrus due to the penetrating character of X-rays. Nevertheless, as the writing was mainly visible by the Pb signal, it is safe to say the Pb concentration calculated by the simulation can be mainly contributed to the ink, and not the underlying papyrus. Taking into account the total area covered by the writing on each fragment and the average sample density, the Pb concentration in the ink is approximately 16 ± 5 μg/cm^2^ for the small and 84 ± 5 μg/cm^2^ for the large fragment.

Based on the work of Kim *et al.*^27^ and Delile *et al.*^28^ it is clear these Pb contents are fairly high to be caused by the contamination of lead in the water (<1.5 mg/L), used as a solvent for the ink, or as a contaminant from a bronze container. Additionally, Cu was found to show no co-distribution with the writing on the papyrus, which would be expected if Pb in the writing was a contaminant from a bronze container (Supplementary Figure S2).

To obtain a better insight in the ink elemental composition, the same μXRF maps were acquired, but zooming on a particular part of the ink, with a smaller beam (down to sub-micrometric probe). It was of particular interest to see if elements, correlated at the millimetre scale, were also correlated at the micro-scale (hence potentially chemically associated). Figure 2 shows the Pb distribution on the large fragment on both macroscopic and microscopic levels, as well as the distribution of Al, P and S at different zoom levels down to the microscopic levels. It is clear from these results that Pb, P and Cl show a co-distribution (Fig. 2 and Supplementary Figures S3 and S4). Even though Al is co-localized with the Pb at the macro level (Fig. 2 and Supplementary Figure S1), it is clear from the micro-scale images Pb is not chemically associated with Al due to their lack of co-distribution at the micro level. This demonstrates that the used ink has a complex composition, with different ingredients of which the distribution is homogeneous at the millimetre scale, but not at the microscopic level. The S signal shows a fairly homogeneous distribution over the papyrus, with occasional hot spots which are only partially co-distributed with Pb (Fig. 2). Taking into account the quantification results, it is thus unlikely that PbS is the main constituent of the papyrus ink.

**Figure 3:**
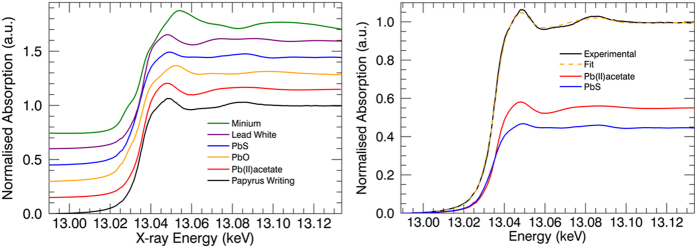
Left: Pb-L3 XANES spectra measured on the writing (black) compared to several other reference compounds. Right: Linear combination fitting showing a clear contribution of lead(II)acetate (red) in the papyrus writing lead content.

Additionally, X-ray absorption near-edge structure (XANES) spectroscopy was performed at the Pb-L_3_ and S-K edges to identify the chemical state and speciation of the Pb and S, by comparison with the XANES spectra of known compounds. Figure 3 displays the Pb-L_3_ XANES of the Pb in the writing, compared to several other reference compounds. It is clear from this image that Pb in the writing is in a state very similar to that of lead(II)acetate. Linear combination fitting of the papyrus data with the reference compound XANES shows there may be a partial contribution of PbS (up to 45%), along with lead(II)acetate. However, it should be noted the PbS XANES post-edge is fairly featureless; PbS contributes to the fit mainly to correct for a small edge shift and shaping of the post-edge feature at 13.085 keV compared to the lead(II)acetate reference. Comparing the papyrus writing Pb-L_3_ XANES curve with those measured for minium in literature^29^,^30^, it is clear no contribution of minium is found in the papyrus writing XANES due to the lack of contribution of the minium white line (~13.055 keV). As such, if minium was used to draft the writing, the compound was not preserved over the centuries.

**Figure 4:**
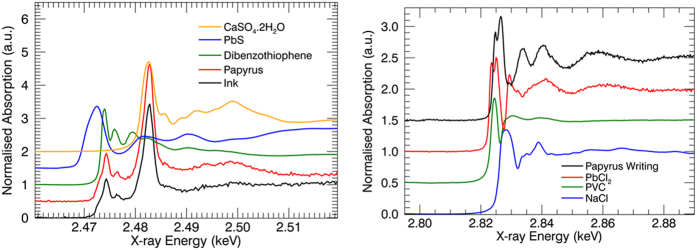
Left: S-K XANES spectra measured on the writing (black) and besides the writing (red) on the papyrus. Several reference compounds’ XANES are displayed for comparison. Right: Cl-K XANES spectra measured on the writing, along with several reference spectra.

As shown in Fig. 4, S-K edge XANES shows complex features, characteristic of a mixture of different species. The primary information is obtained by comparing the signal in and next to the ink. The spectra are quite similar, demonstrating that most of the signal in the area covered by the ink is most probably due to the papyrus behind the ink. The spectra exhibit features similar to those recorded on plants or wood, with both reduced and oxidized sulphur species. Cheah *et al.* discuss the speciation of sulphur in biochar, displaying a S-K edge XANES spectrum very similar to the one obtained from the papyrus and ink^31^. A good match for the reduced S contribution is found with an unknown organic sulphur compound as well as a sulphate, represented here as a dibenzothiophene and Ca-sulphate respectively, as in Cheah *et al.*^31^. More particularly, the presence of PbS in the papyrus writing is not indicated due to the absence of a sulphide peak at 2.4725 keV. A Cl-K edge XANES, measured on the writing, is shown to the right in Fig. 4. Based on comparison to reference materials available (among which PbCl_2_, NaCl, PVC) no match was found. However, comparison to literature shows that the probed Cl compound resembles organochlorine compounds as found in coal fuels^32^. Thus, despite the co-localization of Pb and Cl, they seem not directly chemically bonded in the papyrus ink.

Alternative to the use of a lead pigment, lead could have been introduced as a drier. As mentioned earlier, litharge was used in Antiquity as an oil drier^17^,^18^. As PbO is very prompt to react (e.g. with oil or another reactive), the detection of PbO “as is” is rendered improbable. Previous analyses including among others Fourier-transformed infrared spectroscopy, Raman spectroscopy, gas chromatography coupled with mass spectrometry and pyrolysis, applied to nine black powders found in Pompeii houses (possibly used as writing inks and cosmetics), revealed in eight samples the presence of lipids, of animal and/or plant origin^7^. The FTIR spectrum of one sample shows features characteristic of fatty soaps similar to those found in some Egyptian cosmetics^9^. The hypothesis of the Pb being present as lead carboxylates, consecutive to the use of a Pb-based drier and provided the organic lead species are original, is further strengthened by the Pb-L_3_ XANES as discussed above. Here, μFTIR was not attempted on the papyrus fragments as it would have required destruction of the sample. Therefore, information about organic components is rather limited. The distinction between a lead pigment and a lead drier can only be estimated based on the lead concentration in ink. A survey of several oil paint medium recipes from the XVth to the XXth century states the mass proportions of litharge-oil can vary from 1–4 to 1–16^33^. Using the latter fraction, an estimated writing thickness of 50 μm and an oil density of 0.93 g/mL, a theoretical concentration of 290 μg/cm^2^ of Pb is found. Concentrations obtained through the Monte Carlo quantification are lower (~80 μg/cm^2^). However, the actual ink layer thickness may be thinner than what was estimated here, as well as the proportion of the drier with respect to the “lead-based medium” could be lower. Additionally, the drying medium may have been diluted with other organic binders. It can also be argued that for Pb to originate from a pigment, the Pb concentration should be larger than Pb introduced via a drying agent. As such, the quantified Pb content is an additional argument for Pb originating from an admixture to the ink rather than the main constituent.

Finally, the presence of galena in the writing is discouraged by the S-K edge XANES and the only partial co-distribution of S and Pb at the micro-scale. However, it should be noted that PbS, if originally present, could have oxidized to PbO during the pyroclastic events that carbonized the scrolls. After the scrolls were discovered and exhumed in 1754^1^,^4^,^5^,^6^ they were stored in wooden cabinets, which are known to exhume acetic acid vapours and can slowly react with PbO to form lead(II)acetate or other lead carboxylates^34^,^35^. This latter reaction scheme could also explain the presence of lead(II)acetate, or similar carboxylates, as found by the Pb-L_3_ XANES.

In conclusion, two Herculaneum papyrus fragments were investigated aiming at the non-destructive identification, localization, quantification and speciation of Pb by X-ray based techniques and Monte Carlo simulations. Several hypotheses were postulated towards the origin of the Pb in the scrolls, and assessed using the aforementioned analytical techniques. Based on the Pb concentration and lack of co-distribution with Cu it was determined Pb is not originating from a contaminant in the ink solvent or container. The authors deem it more likely Pb was intentionally added to the ink, either as a pigment or as a drying agent. However, due to the changes the samples have likely undergone during the pyroclastic events and subsequent exhumation, the authors are unsuccessful at providing a definite answer to the question of the origin of Pb in the writing on the scrolls. Nevertheless, the applied analytical methodologies provide important insights in the manufacturing and history of these and similar precious artefacts and should be similarly applied to other papyri, preserved in better conditions. Moreover the found concentrations in these fragments provide important information for optimizing the future tomography experiments on the rolled-up Herculaneum scrolls. Further studies, in particular focusing on the speciation of organic compounds, may provide additional information required to form a definite answer to the origin of Pb in the scrolls, despite the often invasive nature of such techniques.

## Methods

### Description of the samples

The large fragment measures approximately 0.9 × 1.2 cm^2^, whereas the small fragment is approximately 0.5 × 0.8 cm^2^ large. Both samples are originating from unrolled scrolls and have an average thickness of approximately 0.3 mm. Samples were sandwiched in between two 4 μm thick ultralene foils (Spex, Certiprep).

### XRF mapping acquisition

Microbeam measurements were performed at the X-ray microscopy beamline ID21 at the ESRF (Grenoble, France). The primary beam energy was tuned by the use of a Si(111) monochromator. To define the beam spot size on the sample for a general overview mapping, a pinhole (50 μm for the large fragment and 100 μm for the small fragment) was used. An incident beam flux monitoring pin diode was used continuously to correct for intensity variations. An average beam flux of 3.7 × 10^9 ^ph/s and 6.9 × 10^9 ^ph/s was obtained during the measurements on the large and small fragments respectively. The sample to detector chip distance was set to 3 cm and 3.1 cm for the large and small fragments, respectively. Scans were performed by moving the sample through the X-ray beam and acquiring an XRF spectrum at each step (38 × 31 100 μm steps for the small fragment and 160 × 175 50 μm steps for the large one). Acquisition times of 0.26 s and 1 s per step were used for the large and small fragments respectively. High resolution spatially resolved XRF measurements were performed using progressively smaller pixel size (10, 5 and 1 μm), the beam being focused down to ~0.3 × 0.6 μm^2^ using a Kirkpatrick-Baez mirror system.

The microscope is operated under vacuum and samples were placed under an angle of 62° with respect to the primary X-ray beam. The XRF (and scatter) radiation was detected using a Bruker (Germany) XFlash 5100 silicon drift detector (SDD), equipped with a Moxtek AP3.3 polymer window^36^, mounted under 69° with respect to the primary X-ray beam. An additional ultralene foil (4 μm) further covers the detector. XRF spectra were processed using PyMCA^37^ as well as the AXIL and IDL based Microxrf2 software packages^38^,^39^. Pixels contributing to the writing on the papyrus were isolated using K-means clustering routines, as incorporated in the Microxrf2 software^23^.

### XRF quantification

The XRF data was quantified using Monte Carlo based simulations, as calculated by the XMI-MSIM simulation code^24^,^25^,^26^. Sample position and orientation as well as detector position and orientation were calculated based on the information as noted above. A detector active area of 0.8 cm^2^ was supplied without a collimator. Slits sizes were set to the size of the used pinhole. Additionally, the SDD detector crystal was simulated to consist of pure Si (ρ=2.33 g/cm^3^) with a 450 μm thickness. The primary X-ray beam was set to a monochromatic value of 3.5 keV with a 100% polarization in the horizontal plane. A source size equal to the pinhole size with a zero radian divergence was applied. The experimentally used 4 μm ultralene foils were approximated during the simulation as 4 μm Kapton polyimide foils, the composition of which was supplied by the built-in XMI-MSIM catalogue. Due to the small foil thickness and similar energy dependent X-ray absorption this approximation will not have a significant influence on the outcome of the quantification. The Moxtek AP3.3 window, consisting of a thin (<4 μm) polymer foil on top of a 100 μm or more silicon grid^36^, cannot be simulated in the XMI-MSIM program due to its heterogeneous nature. As 100 μm Si is virtually a beam stop to 3.5 keV X-ray photons (approximately 3 × 10^−5^% transmission), regions covered by the Moxtek window grid will not transmit photons to the detector. Provided a 70% open grid area, the Moxtek window Si grid was simulated in the XMI-MSIM program by reducing the initial X-ray beam flux by 30%. The live acquisition time corresponding to the writing sum spectrum was derived from the amount of pixels contributing to the writing and the acquisition time per point, taking into account an average dead time of 5% for the large and 15% for the small fragment. The sample thickness was estimated to be 300 μm and the sample density was calculated to be 0.36 g/cm^3^, based on the dimensions of the approximately cylindrical scroll with a diameter of 5 cm, length of 20 cm and weight of 141 g.

### XAFS

Pb-L_3_ edge XAFS experiments were performed at the EXAFS station of the Dutch-Belgian beam line (DUBBLE, BM26A) at the ESRF (Grenoble, France)^22^. The energy of the X-ray beam was tuned using a Si(111) monochromator operating in fixed-exit mode, with an energy resolution of approximately 1.7 × 10^−4^ at 9.659 keV. Higher harmonics were rejected by using a vertically focusing Pt-coated mirror behind the monochromator. The primary X-ray beam was confined to 1(V) × 3(H) mm^2^ using a slit system and further focused down to approximately 20(V) × 60(H) μm^2^ at the sample position using polycapillary optics. The primary X-ray beam and transmitted beam intensities were monitored using ionization chambers, filled with a gas mixture to absorb approximately 10% and 70% of the beam respectively. The XRF/scattered radiation was detected using a Vortex-EM silicon drift detector.

XAFS energy scans were performed over the Pb-L_3_ edge (calibrated using a metallic Pb foil, setting the maximum of the first derivative to 13.035 keV), starting at E_0_ − 100 eV up to E_0_ + 100 eV. Acquisition times per energy step were chosen based on data statistics and available beam time. Additional XANES acquisitions were performed at ID21, at the sulphur and chlorine K-edges. XANES spectra were recorded in XRF mode (same set-up as above), with a beam of 50, 100 and 0.4 μm diameter. The monochromator was calibrated setting the maximum of absorption at 2.483 keV for CaSO_4_·2H_2_O reference and at 2.828 keV for NaCl reference. XANES were acquired from 2.46 to 2.58 keV with 400 steps of 0.3 eV at S K-edge and from 2.795 to 2.895 keV with 400 steps of 0.25 eV at Cl K-edge. Linear combination fitting of the XANES spectra was performed to identify and quantify mixtures of pure compounds, which were prepared as powder pressed to pellets and measured in transmission mode.

## Additional Information

**How to cite this article**: Tack, P. *et al.* Tracking ink composition on Herculaneum papyrus scrolls: quantification and speciation of lead by X-ray based techniques and Monte Carlo simulations. *Sci. Rep.* **6**, 20763; doi: 10.1038/srep20763 (2016).

## Supplementary Material

Supplementary Information

## Figures and Tables

**Table 1:** Monte Carlo quantification results in w% using the XMI-MSIM software on the sum spectra corresponding to the writing (Ink) on the large (L) and small (S) papyrus fragments, as well as on the surrounding papyrus (Pap).

	C^a^	Na	Al	Si	P	S	Cl	Pb
L-Ink	75.14	0.65	1.00	8.80	0.60	0.01	0.12	0.78
L-Pap	76.56	0.65	0.50	8.80	0.40	0.07	0.08	0.05
S-Ink	77.06	0.35	0.75	8.30	0.35	0.08	0.07	0.15
S-Pap	77.42	0.35	0.60	8.30	0.25	0.07	0.07	0.05

Elements present in the simulation but not shown here are Mg, K, Ca, Ti, Mn, Fe, Ni and Ba.

^a^The C content is determined indirectly by difference and may be a mixture of C,H and O.
