# Supplementary material for: Tracking ink composition on Herculaneum papyrus scrolls: quantification and speciation of lead by X-ray based techniques and Monte Carlo simulations
Source: Sci Rep. 2016 Feb 8;6:20763. doi: 10.1038/srep20763 (PMC4745103; doi:10.1038/srep20763)
Supplement: Supplementary Information [file srep20763-s1.pdf]

Tracking ink composition on Herculaneum papyrus scrolls: quantification and speciation of lead by X-ray based techniques and Monte Carlo simulations

Pieter Tack, Marine Cotte, Stephen Bauters, Emmanuel Brun, Dipanjan Banerjee, Wim Bras, Claudio Ferrero, Daniel Delattre, Vito Mocella, Laszlo Vincze

Supporting Information

Table of Contents

1. Figure S1: large papyrus XRF distribution images 2
2. Figure S2: Cu-K_α distribution on large papyrus fragment 2
3. Figure S3: Pb-Cl correlation plot on micro-scale XRF mapping 3
4. Figure S4: Micro-scale XRF elemental distribution images 3

1. Figure S1: large papyrus XRF distribution images

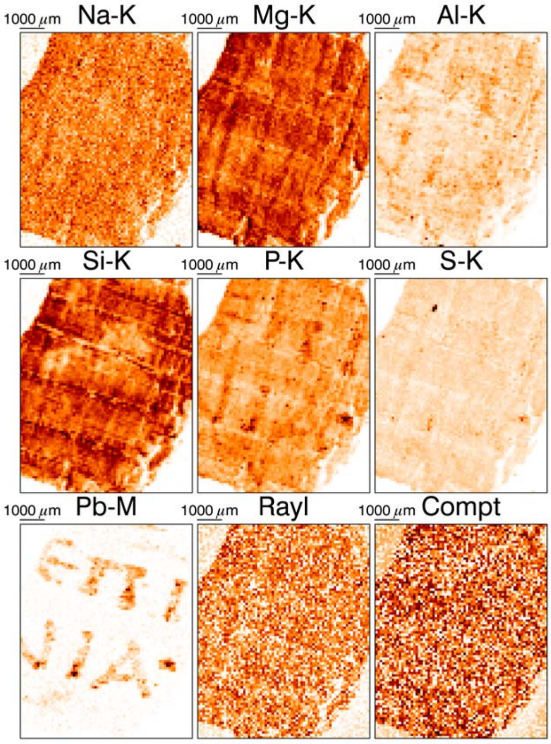

Figure S1. XRF Elemental distribution images of the large papyrus fragment. The writing is visible in the Al, P and Pb signals.

2. Figure S2: Cu-K_α distribution on large papyrus fragment

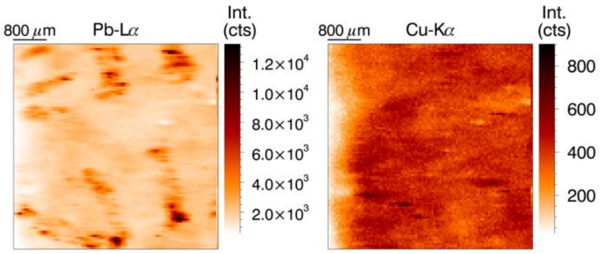

Figure S2. Pb-L_α and Cu-K_α elemental distribution of the large fragment as measured at BM26A using an excitation energy of 13.133 keV.

3. Figure S3: Pb-Cl correlation plot on micro-scale XRF mapping

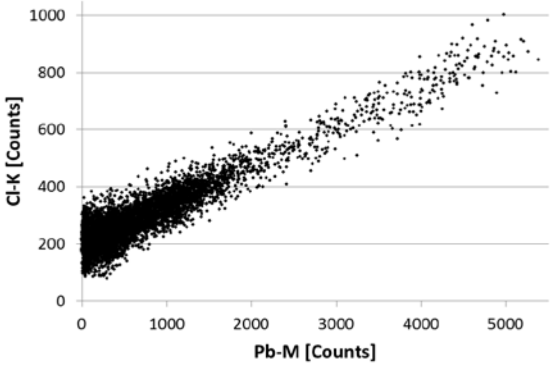

Figure S3. Pb-Cl correlation plot for the micro-scale XRF mapping. A clear correlation is seen between these two elements.

4. Figure S4: Micro-scale XRF elemental distribution images

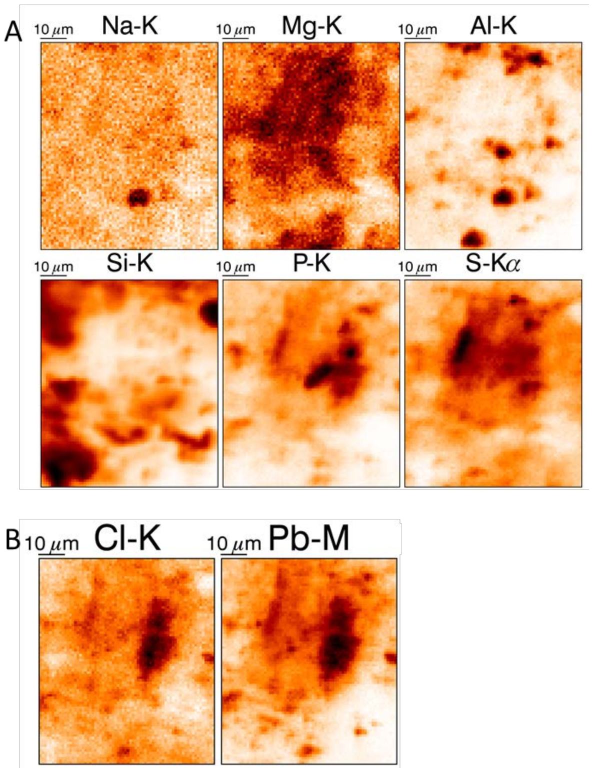

Figure S4. XRF elemental distribution images at the micro-scale (1 µm step size) displaying the co-distribution of P, Cl, Pb and partially S. Mappings in A were measured with an incident beam energy of 2.48 keV, thus not exciting the Pb-M and Cl-K shells electrons. Mappings in B were acquired at E_0 equal 3.00 keV (above the Cl-K and Pb-M shells).
